# Prevalence and predisposing factors for self-reported hypertension in Bhutanese adults

**DOI:** 10.3126/nje.v10i1.25466

**Published:** 2020-03-30

**Authors:** Kinley Wangdi, Tshering Jamtsho

**Affiliations:** 1 Research Fellow, Department of Global Health, Research School of Population Health, The Australian National University, Canberra, Australia; 2 Medical Officer, Phuentsholing General Hospital, Phuentsholing, Chukha Bhutan; 3 PhD Student, School of Demography, ANU College of Arts & Social Sciences, The Australian National University, Canberra, Australia

**Keywords:** Bhutan, hypertension, National Health Survey 2012, risk factors

## Abstract

**Background:**

Bhutan underwent a nutrition transition in the last two decades. Diet has changed from high-fibre, high carbohydrate and low-fat diets to food with high sugar, fat, salt and processed foods. This is further compounded by a sedentary lifestyle. This paper aims to determine the national prevalence of hypertension and study the associated correlates in Bhutanese adults.

**Materials and Methods:**

This study used secondary data from the Bhutan National Health Survey 2012 (NHS, 2012) which was a nationwide survey covering all 20 districts in Bhutan. The dependent variable was self-reported hypertension under medication. Multivariable logistic regression was undertaken to identify independent correlates of hypertension.

**Results:**

The national prevalence of hypertension was 17.4% (5,408). Risk factors for hypertension were female sex, increasing age, occupation of armed forces, manager, technician, service and sales worker, machine operator and monks, diabetes, and feeling worried. Being single was negatively correlated with hypertension. In addition, hypertension is negatively associated with the poverty of the district.

**Conclusion:**

Hypertension was associated with age, being women, occupation with less physical activity, being worried and having diabetes. The preventive measures both at community and healthcare facility-based through cost-effective strategies should target these covariates.

## Introduction

Globally, around one billion over the age of 25 years were diagnosed with hypertension in 2008 [1]. Hypertension is defined as an increase in systolic blood pressure above 140 mmHg and/or diastolic blood pressure equal to or above 90 mmHg [2, 3]. Its prevalence is more in low and middle-income countries compared to high-income countries [3]. It is responsible for 45% of deaths due to heart diseases [4].

Hypertension is caused by both modifiable and non-modifiable risk factors [3]. Modifiable predisposing factors include hyperlipidaemia [5], physical inactivity [6], obesity, kidney disease [7], diabetes, high salt intake [8], alcohol consumption [9, 10], and smoking [11]. Age, ethnic background and gender (female) are important non-modifiable risk factors of hypertension [10]. Effective intervention includes early detection and treatment of hypertension, increased physical activity; reduce salt intake, moderate alcohol consumption, and cessation of smoking.

In the last two decades, Bhutan has undergone a marked nutritional transition [12]. This has resulted in increased disposable income levels and change in food habit with more consumption of saturated fat and salt diet from a traditional low-fat and low-salt diet. Furthermore, people engage in sedentary lifestyle with less physical activity [13]. Rapid urbanisation in last two decade resulted in rural-urban migration and >31% of Bhutanese now live in urban areas. An earlier study reported a national prevalence of hypertension to be 17.1% in 2007 [14], while in the capital city Thimphu it was estimated at 26.0% [15]. However, there is a lack of study on the national prevalence of hypertension and its associated correlates. Therefore, this study aims to determine the prevalence of hypertension and explore the correlates of hypertension in the Bhutanese population. Exploration of such data in Bhutan is anticipated to provide associated correlates for hypertension in a population undergoing the epidemiologic and nutrition transition.

## Methodology

### Study design and participants

A secondary retrospective study was undertaken using the National Health Survey (NHS 2012) data. The Ministry of Health of Bhutan used the Surveillance of non-communicable diseases (STEP) survey guidelines to collect the data in all the 20 districts of Bhutan. The census sample frame for the NHS 2012 was developed using the Population and Housing Census of Bhutan 2005.

### Inclusion criteria

The records of participants above the age of 18 years were extracted from the NHS data for analysis.

### Exclusion criteria

Records with incomplete data were excluded from the final analysis.

### Data collection

The data were obtained from the Ministry of Health data repository. District poverty was obtained from the Bhutan Multidimensional Poverty Index 2012 published online by the National Statistical Bureau (NSB) of Bhutan [16]. The data were then linked to the proportion of hypertension by districts to examine the relationship between hypertension and poverty.

### Sample size calculation

The sample size was calculated for each district using the following formula:

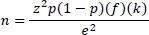

Where: *n* is the required number of households; *z* is the value of the statistic in a normal distribution for a 95% confidence interval (this value is 1.96 and for purposes of calculation it is rounded to 2); *p* is the proportion of households within 1 hour to any health facility; e is the margin of error in calculating *p*, (was 0.05 for this study); *f* is the sample design effect, assumed to be 2.0; and *k* is an anticipated non-response of 5%.

A total of 13,600 households were sampled corresponding to 45,635 individuals aged between 10–75 years.

### Outcome variable

The dependent variable was self-reported hypertension on medication. The participants in this study were the adult population above 18 years. Independent variables were self-reported correlates obtained from the STEPS survey questionnaire.

### Independent variable

Independent variables of interest were age, sex, education level, occupation, urban-rural residence, ever smokers, ever consumed alcohol, vigorous exercise, vegetable servings per day and feeling of worried. This study included all records of participants older than 18 years.

One standard vegetable serving was defined as: (a) 1 cup of raw green leafy vegetable such as spinach, salad greens, etc. (b) ½ cup of other vegetables, cooked or chopped, such as carrots, pumpkin, corn, beans, onion, etc., but excluding tubers such as potatoes. A “standard drink” was quantified through the amount of ethanol in standard glasses of beer, fortified wine such as sherry, wine, and spirits (around 8-13 grams). Worried was defined as a feeling of stress resulting in an inability to sleep at night in the last 12 months. Vigorous activity was defined as any activity that increased heart rate/breathing as in lifting heavy loads, or similar to digging a field for 10 minutes. Hypertension was ascertained by recording medications which lower high blood pressure [17]. These correlates were based on the published literature from earlier studies [18-20].

### Ethical committee approval

The ethical approval for this study was provided by the Research Ethics Board of Health (REBH), Ministry of Health Bhutan via REBH/Approval/2018/041.

### Data management and statistical analysis:

Bivariate and multivariate logistic regression model was used to identify significant correlates. Exploratory variables with a p<0.10 in a bivariate model were entered into the final multivariate model. A value of p<0.05 was considered significant. A goodness-of-fit test was undertaken to measure the adequacy of the final model. Statistical analysis was done in STATA version 15.1 (Stata Corporation, College Station, TX, USA).

## Results

### Characteristics of the study population

A total of 31,066 participants aged 18-75 years were extracted from the NHS 2012. The mean age of the survey participants was 39.3 years (95% confidence interval [CI] 39.2, 39.5) and there were 16,731 (53.9%) women. Twenty-six percent of the participants were in 25-34 age group and 24.6% lived in urban areas. Nearly half of the study population did not have formal education and <1% were diploma or certificate level educated. Two-third were married and 59.8% were farmers. The prevalence of diabetes in the survey was 1.8%. Forty-seven percent and 4.2% drank alcohol and smoked, respectively. Vegetables were consumed by 41.0% in a week. Only 1.0% of participants were always worried. More than 47.4% of the study participants were involved in the physical activities resulting in increased breathing (Table 1).

### Socio-demographic characteristics of hypertensive patients

**Figure 1:**
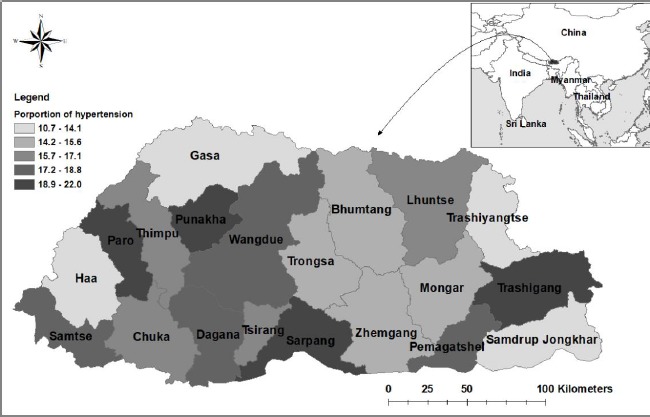
Proportion of hypertension by districts

The national prevalence of hypertension was 17.4% and female constituted 62.3% of the study population. Nearly 11.4% of hypertensive patients were >64 years. Most (76.7%) hypertensive people lived in rural Bhutan. Married participants and those with no formal education made up 81.5% and 59.4% of study participants. Most of the hypertensive patients were farmers, unskilled and clerical workers, 5.7% has concurrent diabetes, and nearly half-consumed alcohol (48.5%), 3.3% were current smokers and 17.1% ever smoked. Only 3.3% did consume vegetables in a typical week, while 40.2%, 29.2% and 27.3% consumed vegetables in 6-7, 4-5 and 1-3 days respectively. More than half of the participants were never worried or felt lonely. Forty-six percent of hypertensive participants engaged in vigorous physical activity (Table 1). The proportion of hypertensive participants ranged from 10.7% to 22.0% with Paro, Punakha and Trashigang districts reporting a higher proportion of hypertension (Figure 1). There was a significant negative association between the proportion of hypertension and district poverty level in the Pearson correlation analysis (r=-0.4831, p=0.0309).

### Factors associated with hypertension

Being female, above 24 years, single and widow, educated up to university or equivalent and non-formal educated, occupation in manager, service and sales worker, machine operator and monk categories, having diabetes, ever smoked, drank alcohol and being worried was associated with hypertension. Eating vegetable servings of 4-5 days per week offered protection against hypertension (Table 2).

**Figure 2:**
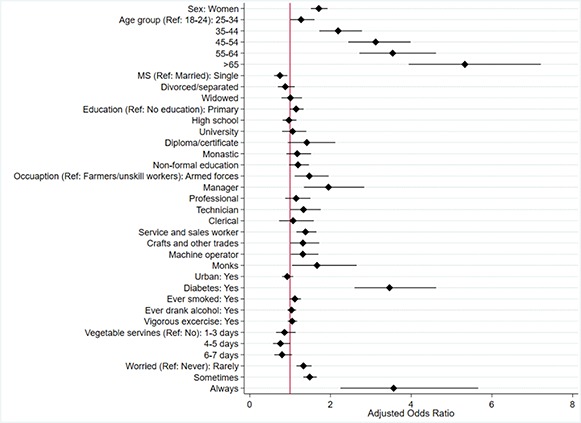
Forest plot adjusted odd ratios of correlates of hypertension

**Figure 3:**
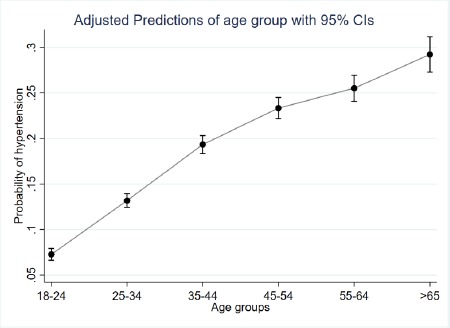
Probability of being hypertensive by age groups

After adjusting for other variables, females were 71% (AOR=1.71; 95% CI: 1.516-1.929) more odds to report hypertension than the male participants. There was a linear increase in hypertension with an increase in age. Participants in age groups of 25-34, 35-44 years, 45-54, 55-64 and over 64 years reported adjusted odds ratio (AOR)=1.27, (95% CI: 1.007-1.605), AOR=2.19 (95% CI: 1.724-2.781), AOR=3.12 (95% CI: 2.441-3.986), AOR=3.54 (95% CI: 2.715-4.616) and AOR=5.33 (95% CI: 3.938-7.213) as compared to participants aged 18-24 years. Being single as compared to married were 25% (AOR=0.75; 95% CI: 0.602-0.931) less likely to be hypertensive. Armed forces (AOR=1.48; 95% CI: 1.113-1.955), manager (AOR=1.95; 95% CI: 1.342-2.836), technician (AOR=1.33; 95% CI: 1.008-1.758), service and sales worker (AOR=1.38; 95% CI: 1.154-1.652), craft and other trades (AOR=1.32; 95% CI: 1.007-1.722), machine operator (AOR=1.31; 95% CI: 1.016-1.701), and monks (AOR=1.67; 95% CI: 1.049-2.649) and were at risk of hypertension in comparison to farmers. Diabetes was a major risk factor for hypertension; participants with diabetes were nearly 3.5 times more likely to be hypertensive compared to those who were not diabetic (AOR=3.46; 95% CI: 2.596-4.617). Factors relating to psychological well-being were at risk of hypertension, such as feeling worried rarely (AOR=1.33; 95% CI: 1.156-1.532), sometimes (AOR=1.48; 95% CI: 1.328-1.660) and always (AOR=3.56; 95% CI: 2.246-5.658) as compared to those participants who were never (Figure 2 & Table 3). The increased probability of hypertension with increasing age with CI 95% has been shown in Figure 3.

## Discussion

The national prevalence of hypertension was 17.4% and the factors associated with hypertension were age, female, being a single, occupation in armed forces, managers, salespersons, technicians and monks, having diabetes and being worried.

### Prevalence of hypertension

Hypertension is one of the top five non-communicable diseases in Bhutan [21]. Uncontrolled and untreated hypertension can lead to a number of complications such as heart failure, renal diseases, ischemic heart disease, pulmonary hypertension and cerebrovascular diseases including stroke [22]. Globally, it is responsible for significant disability and premature deaths [4]. Further, medical expenses in managing hypertension associated complications results are huge [23-25]. Therefore, this nationally representative first epidemiological study is timely and provides much-needed evidence to policymakers of Bhutan to initiate prevention strategies for hypertension.

### Risk factors of hypertension

As in other studies, women were at high risk of hypertension compared to men [26-27]. As the women reach menopause, the protection offered by oestrogen decreases thereby increasing the risk of hypertension [28, 29]. In this study, age was an important non-modifiable risk factor for hypertension which is in conformity with the published literature [30]. The life expectancy of the Bhutanese population has increased from 48 years in 1975 to 69 years in 2008. This increase is due to the introduction of modern health facilities in the 1960s [31, 32]. As population ages, many pre-hypertensive populations will progress to full hypertension in the absence of appropriate preventive measures including screening and pharmacological management.

Diabetes was associated with hypertensive as in other studies [33-34]. This is due to the shared risk factors such as sedentary lifestyle and obesity [35], smoking and alcohol use, and share a common metabolic pathway [36]. Diabetes in general Bhutanese population was around 1.8% [17, 37]. Therefore, national preventive strategies should take a holistic approach incorporating both hypertension and diabetes.

In this study, occupations with less physical activity including monks were at risk of hypertension as compared to jobs that require physical activity. Such findings were reported in other studies from our region [38-39]. The finding that monks are at higher risk of hypertension is important because there is a large number of monks in the monasteries, and most of these monasteries do not engage in regular physical activities since appropriate facilities are not readily available. Preventive measures including regular physical activity need to be initiated, targeting these groups of people.

Hypertension was associated with poor mental health including anxiety [40, 41]. The pathway between anxiety and hypertension is complex. Anxiety can increase blood pressure in the short term. For instance, a phenomenon of increased blood pressure due to the white coat effect associated anxiety is well known [42, 43]. Other pathways that increase blood pressure due to anxiety include a change in the systemic vascular resistance, sympathetic activity, plasma renin activity, the homeostasis model and blood lipids [44-45]. An indirect association between hypertension and anxiety could be related to the characteristics of anxious subjects- who may engage in an unhealthier lifestyle including increased food intake, smoking, alcohol use, and sedentary lifestyle due to stress and anxiety [46].

The risk of hypertension increased with the decreasing level of poverty in this study. This is also evident from the hypertension distribution map of Bhutan. This finding is supported by previous studies from India and Nepal [47, 48], but contrary to other published literature [49, 50]. A plausible reason could be- increased disposable income led to increased buying capacity of fatty, energy-dense processed foods and engage in sedentary lifestyle by the wealthier people.

## Conclusion

The study provides a nationally representative hypertension prevalence of 17.4%. Hypertension was associated with age, being women, occupation with less physical activity, being worried and diabetes. The preventive measures both at community and health facility-based through cost-effective strategies should target these covariates.

### Limitation of the study:

This study has some limitations, firstly, the cross-sectional study design cannot establish a causal relationship. Secondly, hypertension status was self-reported so respondents were subjected to probable recall bias and could not be validated and are generally underreported [51]. However, this recall bias could be minimal since they were those on medication. Thirdly, only hypertensive individuals on medication were included in the study and missed those who were asymptomatic or undiagnosed. Fourthly, self-reported behavioural habits such as vegetable consumption, smoking, alcohol use and physical activity were likely to over or under-reported as a result of social desirability. Finally, the data for this data is rather old and the trends might have changed over time. Nevertheless, it is the first study on the prevalence of hypertension and its correlates in the Bhutanese population using nationally representative data. Therefore, the findings from this study can be used for national preventive strategies in Bhutan.

### Future scope of the study:

Hypertension prevalence in Bhutan was high. The identified risk factors were age, being women, occupation with less physical activity, being worried and diabetes. Therefore, Bhutan should initiate regular screening programmes to detect hypertension in the older population. In addition, it is recommended to undertake prospective studies to monitor the trends of hypertension and identify the associated risk factors.

### What is already known on this topic?

Previous studies on hypertension were done in the capital city Thimphu only. The risk of hypertension increased with age but other risk factors were not explored.

### What this study adds:

This study presents nationally representative hypertension in adult Bhutanese. The identified risk factors in this study can be used to target preventive measures. Further, the findings can be used as a baseline to access the effectiveness of any preventive measures in the country.

## Figures and Tables

**Table 1:** Socio-demographic characteristics of the study population and hypertensive participants

Variables		Total (%)	Hypertensive (%)	Non-hypertensive (%)
**Gender**	Female	16,731 (53.9)	3,371 (62.3)	13,356 (52.1)
	Male	14,335 (46.1)	2,037 (37.7)	12,291 (47.9)
**Age group (years)**	18-24	6,083 (19.6)	442 (8.1)	5,636 (22.0)
	25-34	8,060 (26.0)	1,063 (19.7)	6,995 (27.3)
	35-44	6,172 (19.9)	1,193 (22.1)	4,977 (19.4)
	45-54	5,100 (16.4)	1,190 (22.0)	3,909 (15.2)
	55-64	5,650 (11.4)	1,520 (28.1)	2,639 (10.3)
	65+	2,106 (6.8)	615 (11.4)	1,490 (5.8)
**Urban-rural**	Urban	7,645 (24.6)	1,263 (23.4)	6,376 (24.9)
	Rural	23,420 (75.4)	4,145 (76.7)	19,270 (75.1)
**Education**	No formal education	15,666 (50.6)	3,205 (59.4)	12,458 (48.7)
	Non-Formal Education	2,560 (8.3)	419 (7.8)	2,140 98.4)
	Primary school	3,820 (12.3)	667 (12.3)	3,153 (12.3)
	High school	6,260 (20.2)	689 (12.8)	5,567 (21.8)
	Diploma/Certificate	275 (0.9)	159 (3.0)	222 (0.9)
	University	1,501 (4.8)	53 (1.0)	5,567 (21.8)
	Monastic education	899 (2.9)	199 (3.7)	670 (2.7)
**Marital status**	Single	5,223 (16.8)	363 (6.7)	4,856 (19.0)
	Married	22,985 (74.1)	4,402 (81.5)	18,579 (72.5)
	Divorced/Separated	1,299 (4.2)	242 (4.5)	1,057 (4.1)
	Widowed	1,531 (4,9)	396 (7.3)	1,135 (4.4)
**Occupation**	Clerical/farmer /unskilled	10,061 (66.1)	1,564 (65.4)	8,494 (66.2)
	Army	540 (3.5)	86 (3.6)	454 (3.5)
	Manager and professionals	1,338 (8.8)	202 (8.4)	1,137 (8.9)
	Service and sales worker	3,155 (20.7)	508 (21.2)	2,646 (20.6)
	Monks	134 (0.9)	32 (1.4)	102 (0.8)
**Diabetes**	No	26,132 (98.2)	4,474 (94.3)	21,649 (99.0)
	Yes	491 (1.8)	272 (5.7)	218 (1.0)
**Alcohol intake**	No	16,378 (62.7)	2,784 (51.5)	13,587 (53.0)
	Yes	14,681 (47.3)	2,622 (48.5)	12,056 (47.0)
**Smoking now**	No	29,741 (95.8)	5,232 (96.8)	24,499 (95.5)
	Yes	1,322 (4.2)	176 (3.3)	1,146 (4.5)
**Ever smoked**	No	26,067 (83.9)	4,484 (82.9)	21,573 (84.1)
	Yes	4,999 (16.1)	925 (17.1)	4,074 (15.9)
**Fruit serving per week**	No	16,744 (55.4)	2,818 (53.4)	13,924 (55.8)
	1-3 days	8,352 (27.6)	1,516 (28.8)	6,830 (27.4)
	4-5 days	2,916 (9.6)	518 (9.8)	2,398 (9.6)
	6-7 days	2,239 (7.4)	421 (8.0)	1,819 (7.3)
**Vegetable servings per week**	No	900 (3.0)	178 (3.3)	723 (2.9)
	1-3 days	7,788 (25.7)	1,447 (27.3)	6,340 (25.4)
	4-5 days	9,206 (30.3)	1,549 (29.2)	7,651 (30.6)
	6-7 days	12,420 (41.0)	2,131 (40.2)	10,286 (41.1)
**Anxious/Worried**	Never	18,054 (58.2)	2,701 (50.0)	15,353 (59.9)
	Rarely	4,353 (14.0)	748 (13.8)	3,605 (14.1)
	Sometimes	8,311 (26.8)	1,849 (34.2)	6,462 (25.2)
	Always	317 (1.0)	107 (2.0)	210 (0.8)
**Felt lonely**	Never	19,927 (64.2)	3,045 (56.3)	16,882 (65.9)
	Rarely	4,038 (13.0)	744 (13.8)	3,294 (12.9)
	Sometimes	6,694 (21.6)	1,506 (27.9)	5,188 (20.2)
	Always	380 (1.2)	112 (2.0)	268 (1.0)
**Sports**	No	26,199 (84.4)	4,864 (90.0)	21,335 (83.2)
	Yes	4,849 (15.6)	543 (10.0)	4,306 (16.8)
**Walk or bike**	No	9,091 (29.3)	3,735 (69.1)	7,418 (28.9)
	Yes	21,952 (70.7)	1,673 (30.9)	18,217 (71.1)
**Vigorous activities**	No	16,328 (52.6)	2,900 (53.6)	13,428 (52.4)
	Yes	14,721 (47.4)	2,507 (46.4)	12,215 (47.6)

**Table 2:** Bivariate logistic regression analysis with the correlates of hypertension

Variable		Unadjusted Correlates		
		OR	95% CI	p-value
**Sex**	Male	Ref		
	Female	1.52	1.434, 1.617	<0.001
**Age group**	18-24	Ref		
	25-34	1.94	1.724, 2.175	<0.001
	35-44	3.06	2.722, 3.431	<0.001
	45-54	3.88	3.454, 4.361	<0.001
	55-64	4.37	3.863, 4.938	<0.001
	65+	5.27	4.600, 6.025	<0.001
**Marital status**	Married	Ref		
	Single	0.32	0.282, 0.353	<0.001
	Divorce/separated	0.97	0.838, 1.116	0.648
	Widow	1.47	1.305, 1.656	<0.001
**Education**	No education	Ref		
	Primary	0.82	0.749, 0.901	<0.001
	High	0.48	0.440, 0.525	<0.001
	University or equivalent	0.46	0.391, 0.548	<0.001
	Diploma/certificate	0.93	0.686, 1.256	0.629
	Monastic education	1.11	0.941, 1.302	0.219
	NFE	0.76	0.681, 0.852	<0.001
**Occupation**	Farmers	Ref		
	Army	1.01	0.794, 1.277	0.956
	Managers	1.53	1.116, 2.090	0.008
	Professionals	0.84	0.699, 1.004	0.055
	Technicians	0.86	0.688, 1.081	0.199
	Clerical	0.78	0.544, 1.119	0.177
	Service and sales workers	1.16	1.016, 1.321	0.028
	Other trades	1.02	0.805, 1.299	0.855
	Machine operators	0.73	0.582, 0.914	0.006
	Monks	1.7	1.140, 2.534	0.009
**Urban-rural**	Rural	Ref		
	Urban	0.95	0.889, 1.017	0.142
**Diabetes**	No	Ref		
	Yes	6.04	5.038, 7.235	<0.001
**Ever smoked**	No	Ref		
	Yes	1.09	1.009, 1.181	0.028
**Ever drank alcohol**	No	Ref		
	Yes	1.06	1.001, 1.126	0.047
**Vigorous exercise**	No	Ref		
	Yes	0.95	0.896, 1.008	0.89
**Vegetable servings days per week**	0	Ref		
	1-3	0.93	0.784, 1.110	0.433
	4-5	0.83	0.696, 0.984	0.032
	6-7	0.85	0.714, 1.005	0.056
**Worried**	Never	Ref		
	Rarely	1.18	1.079, 1.288	<0.001
	Sometimes	1.63	1.523, 1.738	<0.001
	Always	2.92	2.308, 3.698	<0.001

**Table 3:** Multivariate logistic regression analysis with risk factors for hypertension

Variables		Adjusted Correlates		
		OR	95% CI	P value
**Sex**	Male	Ref		
	Women	1.71	1.516, 1.929	<0.001
**Age group**	18-24	Ref		
	25-34	1.27	1.007, 1.605	0.044
	35-44	2.19	1.724, 2.781	<0.001
	45-54	3.12	2.441, 3.986	<0.001
	55-64	3.54	2.715, 4.616	<0.001
	>65	5.33	3.938, 7.213	<0.001
**Marital Status**	Married	Ref		
	Single	0.75	0.602, 0.931	0.009
	Divorced/separated	0.88	0.696, 1.114	0.29
	widowed	1.01	0.785, 1.295	0.949
**Education**	No education	Ref		
	Primary	1.15	0.991, 1.335	0.066
	High school	0.97	0.814, 1.155	0.731
	University	1.06	0.803, 1.401	0.68
	Diploma/certificate	1.41	0.942, 2.121	0.095
	Monastic	1.18	0.910, 1.519	0.215
	Non-formal education	1.19	0.973, 1.466	0.09
**Occupation**	Farmer	Ref		
	Armed forces	1.48	1.113, 1.955	0.007
	Manager	1.95	1.342, 2.836	<0.001
	Professional	1.15	0.878, 1.504	0.312
	Technician	1.33	1.008, 1.758	0.044
	Clerical	1.07	0.726, 1.587	0.724
	Service and sales worker	1.38	1.154, 1.652	<0.001
	Craft and other trades	1.32	1.007, 1.722	0.044
	Machine operator	1.31	1.016, 1.701	0.037
	Monks	1.67	1.049, 2.649	0.031
**Urban**	No	Ref		
	Yes	0.93	0.800, 1.077	0.326
**Diabetes**	No	Ref		
	Yes	3.46	2.596, 4.617	<0.001
**Ever smoked**	No	Ref		
	Yes	1.12	0.985, 1.268	0.085
**Ever drank alcohol**	No	Ref		
	Yes	1.04	0.934, 1.148	0.509
**Vigorous exercise**	No	Ref		
	Yes	1.05	0.939, 1.173	0.398
**Vegetable serving in a week**	No	Ref		
	1-3 days	0.86	0.651, 1.136	0.288
	4-5 days	0.76	0.577, 1.002	0.052
	6-7 days	0.80	0.608, 1.049	0.106
**Worried**	No	Ref		
	Rarely	1.33	1.156, 1.532	<0.001
	Sometimes	1.48	1.328, 1.660	<0.001
	Always	3.56	2.246, 5.658	<0.001
